# Isolation and Characterization of Phages Infecting *Bacillus subtilis*

**DOI:** 10.1155/2015/179597

**Published:** 2015-07-26

**Authors:** Anna Krasowska, Anna Biegalska, Daria Augustyniak, Marcin Łoś, Malwina Richert, Marcin Łukaszewicz

**Affiliations:** ^1^Department of Biotransformation, Faculty of Biotechnology, University of Wroclaw, Fryderyka Joliot-Curie 14a, 50-383 Wroclaw, Poland; ^2^Department of Pathogen Biology and Immunology, Institute of Genetics and Microbiology, University of Wroclaw, Przybyszewskiego 63-77, 51-148 Wroclaw, Poland; ^3^Department of Molecular Biology, University of Gdańsk, Kładki 24, 80-822 Gdańsk, Poland; ^4^Institute of Physical Chemistry, Polish Academy of Sciences, Kasprzaka 44/52, 01-224 Warsaw, Poland; ^5^Phage Consultants, Tenisowa 10/5, 80-180 Gdańsk, Poland; ^6^Laboratory of Electron Microscopy, Faculty of Biology, University of Gdańsk, Kładki 24, 80-822 Gdańsk, Poland

## Abstract

Bacteriophages have been suggested as an alternative approach to reduce the amount of pathogens in various applications. Bacteriophages of various specificity and virulence were isolated as a means of controlling food-borne pathogens. We studied the interaction of bacteriophages with *Bacillus* species, which are very often persistent in industrial applications such as food production due to their antibiotic resistance and spore formation. A comparative study using electron microscopy, PFGE, and SDS-PAGE as well as determination of host range, pH and temperature resistance, adsorption rate, latent time, and phage burst size was performed on three phages of the *Myoviridae* family and one phage of the *Siphoviridae* family which infected *Bacillus subtilis* strains. The phages are morphologically different and characterized by icosahedral heads and contractile (SIOΦ, SUB*ω*, and SPO*σ* phages) or noncontractile (AR*π* phage) tails. The genomes of SIOΦ and SUB*ω* are composed of 154 kb. The capsid of SIOΦ is composed of four proteins. Bacteriophages SPO*σ* and AR*π* have genome sizes of 25 kbp and 40 kbp, respectively. Both phages as well as SUB*ω* phage have 14 proteins in their capsids. Phages SIOΦ and SPO*σ* are resistant to high temperatures and to the acid (4.0) and alkaline (9.0 and 10.0) pH.

## 1. Introduction

Recent investigations show that bacteriophages could be used as a means of controlling food-borne pathogens [1–3]. New phages are therefore being isolated and characterized [4, 5]. One of the bacterial genera with the highest prevalence in food industry, which could potentially be controlled by bacteriophages, is* Bacillus*, which belong to the heterogeneous group of Gram-positive, endospore-forming, facultative anaerobic bacteria [6, 7]. Despite the numerous advantages of* Bacillus* species in industry applications, they may also cause damage. The pathogenicity of* Bacillus anthracis* for mammals is well known, and other* Bacillus* species infections have been documented since the beginning of the last century [8, 9].* Bacillus cereus* is considered a pathogenic species causing local infections, bacteremia, septicemia endocarditis and pericarditis, and infection of the central nervous system and respiratory tract [10–15].* Bacillus cereus* is the main factor in food poisoning resulting from toxin production [16]. Beside the well-known pathogenic* Bacillus* species, recent studies have detected the production of enterotoxins and emetic toxin by* B. subtilis, B. pumilus*, and* B. licheniformis*, resulting in food-borne illnesses [17–19].* Bacillus subtilis* contaminated 25% of samples of food products in The Netherlands (milk, yeast, flour, pasta products, cocoa, chocolate, bakery products, meat products, herbs, and spices) [20] and 12% of raw materials, dough and bread samples in South Africa [21]. Moreover,* B. subtilis* spore contamination has been found in wheat flour, ropy bread [22], and gelatin [23].

The removal of* Bacillus* contamination is limited due to its resistance to physicochemical decontaminations methods. Usually the food industry uses physical methods of sterilization or disinfection such as* uv*, gamma radiation, or high temperature, but, unfortunately, they are often not effective against the highly resistant* Bacillus* spores [24, 25]. One option for* Bacillus* contamination treatment could be phagotherapy [26].

Bacteriophages are the largest group of viruses [27, 28]. They are very useful for people in phage therapy [29]. On the other hand, preventive phage therapy may eliminate pathogenic bacteria from food products. Phages are host-specific and infect only specific species or strains [1] with a few exceptions [30]. Although investigations have shown that bacteriophages can aid in successful eradications of food-borne pathogens such as* Listeria monocytogenes, Escherichia coli, Salmonella typhimurium*, or* Campylobacter* [1, 31], there are few commercial products, such as LISTEX P100, which have received the GRAS status [32]. Thus, it is necessary to determine the criteria for the application of phages in the control of bacteria in food and therefore all newly isolated phages with the potential application in antibacterial therapy should be characterized in detail. Beside using isolated phages as decontamination agents, they could be used in bacterial detection and serotyping or strain improvement recently described by Petty et al. [33]. However, the commercial application of bacteriophages requires the optimization of culture conditions and detailed determination of the physical and chemical factors influencing phage viability [34, 35]. The first attempts to control* Bacillus subtilis* infections by phages were carried out by Ackermann et al. [36]. The results showed an extreme heterogeneity of* B. subtilis* strains. Ackermann's phagotype system was used by Inatsu et al. [37] for the differentiation of* B. subtilis* (natto) and other* B. subtilis* strains isolated from fermented soybean foods [38].

The aim of this work was to isolate and characterize* Bacillus* bacteriophages in view of their potential application in phagotyping, strain improvement, and eradication of unwanted* Bacillus* strains in food industries. We have isolated four bacteriophages with different specificity to 20* B. subtilis* strains. Detailed data on their morphology, thermal and pH stability, genome size, and capsid protein mass are described for bacteriophages specified for four representative* B. subtilis* strains.

## 2. Materials and Methods

### 2.1. *Bacillus* Strains and Bacteriophages

The origins of bacteriophages used in this study as well as their host strains are shown in Table 1.* Bacillus* strains 10 (isolated from soil), SWV215 [39], B3 [40], and ATTC6633 [41] were used for detailed investigation (Table 1).* Bacillus* strains were maintained as frozen stocks at −80°C in 1.5%, nutrient broth (Biocorp, Poland) with 2% agar-agar (BioShop, Canada Inc) supplemented with 15% (v/v) of glycerol. For experiments, the strains were cultured on the nutrient agar and then cultured overnight in a liquid nutrient broth. The* Bacillus subtilis*-specific bacteriophages SUB*ω*, SPO*σ*, SIOΦ (Acc. number KC699836), and AR*π* were isolated from soil supplemented with a liter of* Bacillus subtilis* cultures. After a week, soil samples (1 g) were suspended in water, shaken (200 rpm/30 min), and centrifuged (4500 rpm/10 min), and the supernatant was filtered through a polyethersulfone (PES) 0.22 *μ*m Millipore filter. Phage propagation was performed as follows: 45 mL of filtered soil samples and 5 mL culture of* Bacillus* strain grown overnight in nutrient broth were added to 20 mL of fresh nutrient broth and incubated for 24 h at 37°C. The suspension was centrifuged (4500 rpm/10 min) and the supernatant was filtered through a PES 0.22 *μ*m Millipore filter. The bacteriophage titer in the filtrate was assessed using the double-agar layer technique of Adams [42].

Phage stocks were prepared in nutrient broth and then stored at 4°C.

### 2.2. Electron Microscopy

A high-titer phage lysate filtered through a PES 0.22 *μ*m Millipore filter was centrifuged at 25,000 ×g for 60 min, and the pellet was washed twice in ammonium acetate (0.1 M, pH 7.0). A portion of the resuspended sediment was deposited on carbon-coated Formvar films, stained with 1% uranyl acetate for 30 s, and examined in a Philips CM 100 (Philips CM 100, Japan) transmission electron microscope at 80 kV with 39,000x magnification. The phage size was determined from the average of five to seven independent measurements using T4 phage tail (114 nm) as the magnification control.

### 2.3. Thermal Treatment

The thermal resistance of all bacteriophages was determined at 23, 50, 60, 70, 80, 90, and 100°C in a temperature-controlled thermo-block (Labnet International. Inc.), at 121°C in an autoclave, at 4°C and −20°C in a standard refrigerator, and at −80°C in a low temperature freezer (REVCO Sanyo, Japan). Equal volumes of phage (10^7^ pfu/mL in sterile water) were incubated for 1, 2, 5, 10, 15, 30, 60, and 180 min at 4°C and then placed in an ice bath or thawed. Samples were assayed to determine surviving pfu using the double-agar layer technique [42].

### 2.4. pH Treatment

The influence of pH on the bacteriophages was assayed in CP (citrate-phosphate) buffer (0.2 M Na_2_HPO_4_/0.1 M citrate) within the pH range of 2.0 to 8.0 and in carbonate buffer (0.2 M H_2_CO_3_/0.2 M NaHCO_3_) within the pH range of 9.0 to 11.0. Experiments were carried out at room temperature (25°C) for 1 and 6 h. Samples were assayed to determine pfu using the double-agar layer technique [42].

### 2.5. Host Range Determination

The host range of bacteriophages was assayed according to Yang et al. [43] as follows: 10^8^ bacterial cells were mixed with molten 0.6% agar and the mixture was poured on 2% solid agar to make double layer agar plates. After solidification, 10 *μ*L of bacteriophage (>10^6^ pfu/mL) was spotted on each plate with each of the different* Bacillus* strains, incubated, and the presence of lysed plates was examined to determine host range.

### 2.6. Adsorption Rate, Latent Time, and Phage Burst Size

Experiments were performed as described previously [42, 44, 45] with slight modifications. Equal volume of phages (10^6^–10^7^ pfu/mL) was added to 0.9 mL of overnight cultures of host bacterial species and incubated at 37°C for 5 min. After incubation, the mixture was diluted to 10^−5^ and filtered through a PES 0.22 *μ*m Millipore filter. The number of free phages in the filtrate was determined in duplicate, using the double-agar layer method.

To determine the phage latent time and burst size a one-step experiment was carried out according to the previous descriptions [46] with modifications. To 0.9 mL of overnight cultures of host bacteria was added 0.1 mL of the bacteriophage suspension. The bacteria were allowed to adsorb the bacteriophages for 5 min at 37°C; the mixture was diluted to 10^−5^ or 10^−4^ and further incubated for 135 min at 37°C. Samples were taken at 10 min intervals and phage titer was determined by the double layer-agar plate method. Burst size was calculated as the ratio of the final amount of liberated phage particles to the initial number of infected bacterial cells during the latent period [42].

### 2.7. Phage DNA Extraction

Phage particles were partially purified by PEG precipitation [47]. High titer suspensions (10^10^ pfu/mL) of filtered phage lysate were mixed with NaCl (1 M final conc.) and incubated on ice for 1.5 h. Then PEG 8000 was added to a final concentration of 10% and the mixture was incubated on ice for 2.5 h. This was followed by the centrifugation of precipitated phages (10 000 g, 4°C, 20 min). Pellets were suspended in 10 mM TE buffer (pH 8.0) or TM buffer (10 mM Tris-HCl, 100 mM NaCl, 10 mM MgCl_2_; pH 7.4). The residual PEG and bacterial debris were next removed by gentle extraction for 30 s with an equal volume of chloroform, followed by centrifugation at 3000 ×g, 4°C for 15 min.

### 2.8. Pulsed-Field Gel Electrophoresis PFGE

Determination of bacteriophage genome size by PFGE was performed according to Lingohr et al. [48]. Purified phage particles in 10 mM TE buffer (pH 8.0) were incorporated in 1 : 1 proportion into 2% low-melting agarose in 10 mM TE. The solid gel plugs were incubated for 2.5 h at 54°C in 1 mL of phage lysis buffer containing 50 mM pH 8.0 Tris-HCl, 50 mM EDTA, 1% (w/v) SDS, and 100 *μ*g/mL (final conc.) of proteinase K (phages SUB*ω*, SPO*σ*, SIOΦ) or 1 mg/mL of proteinase K (phage AR*π*). The plugs were washed at least 4 times in 10 mM Tris/EDTA buffer (TE, pH 8.0) and pulsed-field gel electrophoresis (PFGE) was performed (Bio-Rad, UK) in 1% agarose in 0.5X TBE buffer (pH 8.3). The PFGE parameters were as follows: 0.5X TBE buffer; 6 V/cm, initial switch time 1 s; final switch time 15 s; run time 16 h; temperature 12°C. Gels were stained with ethidium bromide followed by destaining in miliQ water. The genome size was determined with BioRad Quantity One software, using the Low Range PFG marker (New England BioLabs, UK) as a standard.

### 2.9. Preparation of Phage Structural Proteins and SDS-PAGE

Concentrated phage particles in TM buffer were extracted with chloroform (1 : 1 v/v) and, after gentle mixing, were centrifuged at 3000 ×g, 4°C for 15 min. Partly purified phage particles in the aqueous phase were pelleted by ultra-centrifugation (Beckman Coulter) at 50 000 rpm at 4°C for 60 min. The pellets were resuspended in 1X Laemmli loading buffer (62.5 mM Tris-HCl, 2% SDS (w/v), 5% *β*-mercaptoethanol (v/v), 10% glycerol (v/v), 0.04% (w/v) bromophenol blue; pH 6.8), boiled for 5 min, and separated on the SDS-PAGE 12% gel. All gels were run at 40 mA using a mini Protean apparatus (BioRad) and Laemmli buffer. Gels were stained with Gel Code Blue stain reagent (Thermo Scientific). Biorad Quantity One software was used for the molecular analysis of the phage structural proteins based upon a PageRuler prestained protein ladder plus (Fermentas).

### 2.10. Statistical Analysis

All experiments were performed at least in triplicate. For the statistical analysis of data obtained, Microsoft Excel 2007 (SD and *t*-test) and R (ANOVA) programs were applied. Standard deviation was included in figures. Statistical significance was determined using Student's *t*-test. The significance level was set at *P* < 0.05.

## 3. Results

### 3.1. Phage Isolation

*Bacillus subtilis*-specific bacteriophages were isolated from soil inoculated with four distinct (isolated from different locations)* B. subtilis* strains: 10, B3, ATTC 6633, and SWV215. All isolated phages formed clear plaques with the strains used for isolation. Phage titers ranged from 10^10^ for SUB*ω* and SPO*σ* to 10^13^ for SIOΦ (Table 2).

### 3.2. Phage Typing

Twenty* Bacillus subtilis* strains were used to determine the specificity of the four isolated bacteriophages: SUB*ω*, SPO*σ*, SIOΦ, and AR*π* (Table 1). Phage SUB*ω* infected all* Bacillus* strains tested with the exception of* B. subtilis* 10. For* B. subtilis* 168, B20, KT20, B24, and PCM 486 the resulting plaques were hazy. Phage SPO*σ* did not form plaques with* B. subtilis* 10 and KT20 strains, but formed distinct clear plaques with all other strains. Phage SIOΦ formed plaques with* B. subtilis* strains 168, 10, KT20, and PCM 2226 but clear plaques were observed only with* Bacillus* strains 10 and PCM 2226. Phage AR*π* did not form plaques with strains ATCC 6633, SWV215, 10, PCM1938, PCM 2226, PCM 2189, and PCM 2224. Clear plaques were observed for P22 and 172 strains and for B3 and its mutants (Table 1).

### 3.3. Phage Morphology

**Figure 1:**
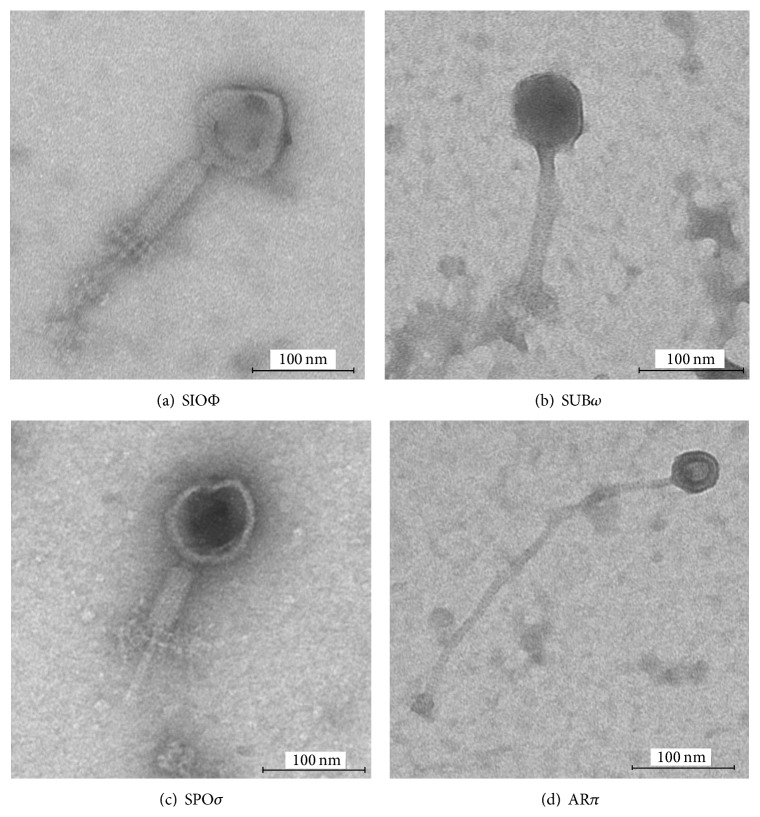
Transmission electron microscopic images of (a)* B. subtilis* 10 phage SIOΦ, (b)* B. subtilis* ATCC 6633 phage SUB*ω*, (c)* B. subtilis* SWV215 phage SPO*σ*, and* B. subtilis* B3 phage AR*π*.

The phages belonged to two morphotypes. Three phages (SIOΦ, SUB*ω*, and SPO*σ*) were classified to* Myoviridae* because of the appearance of icosahedral heads and contractile tails with necks, collars, and base plates (Figures 1(a), 1(b), and 1(c)). Phage head and tail length also indicated relationship to the* Myoviridae* (Table 3).

Bacteriophage AR*π* had icosahedral heads and noncontractile, long tails (Figure 1(d), Table 3) and is thus morphologically similar to phages belonging to* Siphoviridae* family.

### 3.4. Thermal and pH Stability Test

The thermal stability test was carried out to determine the heat resistance of tested phages at pH 7.0. All phages were stable after 180 min at −80°C, −20°C, 4°C, 23°C, 30°C, 37°C, and 50°C, and all phages retained 100% infection activity after a 1 min incubation at 90°C, 100°C, and 121°C (data not shown). An ANOVA test was done and the interaction between the types of phage tested parameters was found statistically important. Thus, it can be said that the inactivation rate of the phages varies for different types of phages.

**Figure 2:**
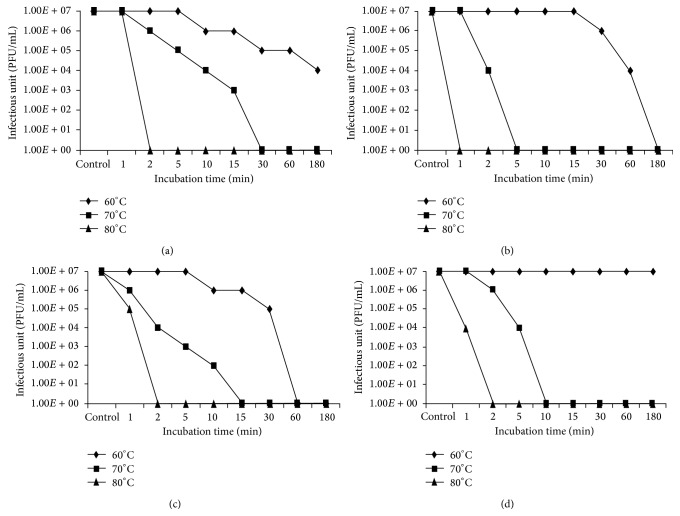
Thermal stability test of phages: (a) SIOΦ, (b) SUB*ω*, (c) SPO*σ*, and (d) AR*π*.

Phage SIOΦ lost total activity after 2 min at 80°C, 30 min at 70°C, and at 60°C only 1% of original activity was observed after a 3 h incubation period. Phage SUB*ω* lost its infective ability after 1 min at 80°C, 5 min at 70°C, and 180 min at 60°C. Phage SPO*σ* was inactive after 2 min at 80°C, 15 min at 70°C, and 60 min at 60°C, respectively. Phage AR*π* lost its activity after 2 min at 80°C, 10 min at 70°C, and 3 hours at 60°C (Figures 2(a), 2(b), 2(c), and 2(d)).

**Figure 3:**
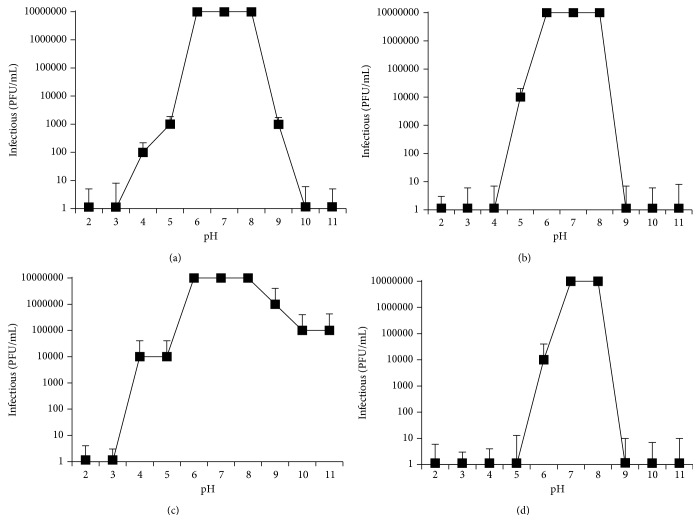
pH stability test of phages: (a) SIOΦ, (b) SUB*ω*, (c) SPO*σ*, and (d) AR*π*. Data are the means from three independent experiments +SD.

Optimal pH was determined by testing the stability of phages at different pH after 60 min (data not shown) and 6 h of incubation at room temperature (25°C). After 1-hour incubation at pH 2.0 all tested phages lost infective ability. Statistically significant differences between tested phages were found. Phage SPO*σ* seems to be extremely stable in the pH range 6.0–8.0; after a 6 h incubation the SPO*σ* activity was reduced from 10^7^ to 10^4^ pfu/mL at pH 3.0 and pH 4.0 (Figure 3(c)). At alkaline pH of 10.0 and pH 11.0 the activity was reduced by one order of magnitude (Figure 3(c)).

Bacteriophage AR*π* was the most sensitive to acid and alkaline conditions and only showed 100% activity at pH 7.0 and pH 8.0 (Figure 3(d)). Similar results were observed after only 1 hour incubation (data not shown).

Phages SIOΦ and SUB*ω* had 100% activity in the pH range of 6.0–8.0 and SIOΦ had reduced activity at pH 4.0, 5.0, and 9.0 from 10^7^ to 10^2^, 10^3^, and 10^3^, respectively (Figures 3(a) and 3(b)).

Phage SIOΦ was the most active in the adsorption (50%) to the* B. subtilis* 10 strain. The one-step growth curve of SIOΦ indicated that the latent period was 55–65 min and the estimated burst size was ~74 phage particles per infected bacterial cell (Table 4). Phage SPO*σ* was less active. It was adsorbed by only 7.5% of the* B. subtilis* SWV215 strain. The latent period of SPO*σ* was 75–85 min and the estimated burst size was ~23 phage particles per infected bacterial cell (Table 4). The latent periods of phages SUB*ω* and AR*π* were the same as that of phage SIOΦ, but the adsorption was lower (12%) and growth times were shorter (30 min). A difference between phages SUB*ω* and AR*π* was observed in their burst size: for SUB*ω* it was only ~8 phage particles per infected bacterial cell while for AR*π* it was ~37 phage particles (Table 4).

### 3.5. Phage Genome Size Estimated by PFGE

**Figure 4:**
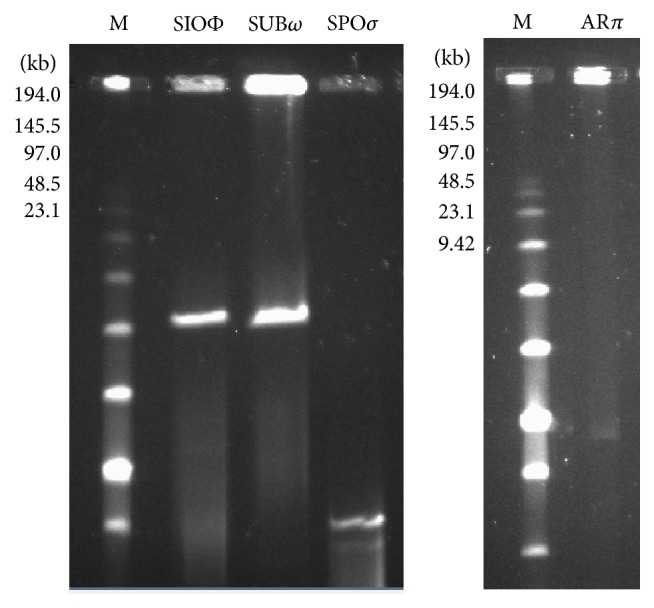
Pulsed-field gel electrophoresis of undigested phage DNAs. Genome sizes were estimated by using Low Range PFG marker (M).

The size of the intact genomic DNA of the four phages in this study was estimated by pulse-field electrophoresis (PFGE). For phages SIOΦ, SUB*ω*, and SPO*σ* belonging to* Myoviridae* family the size of their genomes was, respectively, 154, 154, and 25 kbp (Figure 4). The genome size of Ar*π*, the only phage from* Siphoviridae* family, was 40 kbp (Figure 4). The determined genome size values are comparable with values estimated by ICTV for the indicated phage families.

### 3.6. Protein Composition

**Figure 5:**
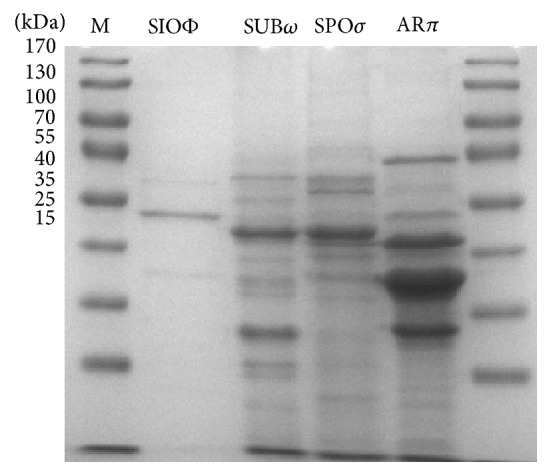
SDS-polyacrylamide gel electrophoretic analysis of indicated virion proteins estimated by using PageRuler prestained protein ladder plus (M).

SDS-PAGE was used to determine the content of structural proteins of phage particles. Virions of phages SIOΦ, SUB*ω*, and SPO*σ* contained structural proteins with predominant protein bands corresponding, respectively, to ~52 kDa (SIOΦ), ~45, 31 kDa (SUB*ω*), and 45 kDa (SPO*σ*) (Figure 5). Phage AR*π* contained at least 14 structural proteins and was found to produce three major protein bands at molecular masses of approximately 42, 37, and 31 kDa (Figure 5).

## 4. Discussion

*Bacillus* has a high prevalence in nature and ability to form heat-resistant endospores; hence it is difficult to eradicate it from, for example, fermented food, and the constant presence of beneficial microorganisms is critical to maintaining the expected food quality. Bacteriophages are host-specific natural enemies of bacteria and the efficient eradication of bacterial pathogens with the use of phages is an effective way to control harmful bacteria [2, 49].

Bacteriophages may have distinct applications, such as biocontrol (eradication) of unwanted species (strains), improvement (selection) of strains used for production, and strain typing. All these applications depend on phage specificity [50]. Both a wide and a narrow host range may be useful. While a rather narrow host range will enable distinction of closely related strains in strain typing, a rather wide host range will be beneficial for biocontrol. In comparison to biocontrol, application of bacteriophages for improvement and selection of industrial strains has been poorly described. In fact, it is well known that in a large-scale production phage infection may lead to the collapse of the production, as it was in the case of acetone-butanol fermentation [51]. Thus, in industrial practice either various strains are used every month or a cocktail of strains is used for inoculum. Rotation of strains with various resistance decreases the risk of accumulation of bacteriophages infecting producer strain in industrial environment [52].

We found four bacteriophages with different specificity toward twenty* Bacillus subtilis* strains (Table 1). The classification of bacteriophages is in most cases based on morphological criteria and rarely on molecular data, for example, nucleotide sequence homology [53]. Considering the morphology, 96% of all bacteriophages belong to the order* Caudovirales* and this order is subdivided into three families: the* Myoviridae* (25%), characterized by a contractile tail; the* Siphoviridae* (61%) with a noncontractile tail; and the* Podoviridae* (14%) with a short, noncontractile tail [54, 55]. In 2009 the International Committee on the Taxonomy of Viruses (ICTV) proposed the creation the* Myoviridae* subfamily* Spounavirinae* with genera of SPO1-like viruses and Twort-like viruses and eight phage species [56]. Recently, Klumpp et al. [57] proposed a revision of current taxonomic organization of the family* Myoviridae* and evaluated new members of this group. In this work we isolated three phages, SIOΦ, SUB*ω*, and SPO*σ*, which morphologically belong to the* Myoviridae* family and phage AR*π* which is classified in the* Siphoviridae* family (Figure 1). The genomes of SIOΦ and SUB*ω* are 154 kbp each and are similar to the other phages of* Bacillus subtilis* such as SPO1 (145.7 kbp), SP8, SP82G, H1, 2C, Φe, or Φ25 (~150) [57]. SIOΦ has only four capsid proteins like the ΦH-like viruses; however, viruses of the ΦH type have a mere 59 kbp genome [58]. Bacteriophage SPO*σ* has a genome size of 25 kbp and ~14 capsid proteins and these features allow it to fall into the group of P2-like or Mu-like viruses but, unfortunately, viruses in this class have Gram-negative bacteria as hosts [58]. AR*π* phage genome is sized 40 kbp and it has ~14 capsid proteins. On the basis of these characteristics it can be classified as N_15_-like virus but the very long tail (342 nm) excludes it from the known classification [58]. The bacteriophages with the longest tails in* Siphoviridae* family belong to ΦM_1_-like viruses (210 nm) and their tail is terminated with a knob as in AR*π* phage.

The use of bacteriophages in biotechnological processes requires the knowledge of their characteristics such as the host range, latent period, growth time, or the resistance to stress conditions, for example, different temperatures or pH. Temperature plays a crucial role in bacteriophage survival, capacity for attachment, and the length of the latent period [59]. SIOΦ, SUB*ω*, SPO*σ*, and AR*π* bacteriophages retain a stable activity following 3 hours at temperatures ranging from −80°C to 50°C (Figure 2). Phages SIOΦ, SPO*σ*, and AR*π* proved to be resistant to high temperatures and all of them survive after 2 min at 80°C, 5 min or longer at 70°C, and 180 min (except for 60 min for SPO*σ*) at 60°C (Figure 2). In general, members of* Myoviridae* and* Siphoviridae* families are considered to be resistant to large temperature fluctuations [35].

Acidity and alkalinity of the environment are other important factors influencing phage stability. SIOΦ and SPO*σ* phages are the most resistant to acid (4.0) and alkaline (9.0 and 10.0) pH; AR*π* phage is the least resistant to pH fluctuations. The optimum pH values for all investigated phages were 7.0 and 8.0 (Figure 3). Lasobras et al. [60] have suggested that members of* Siphoviridae* family are the most resistant to adverse conditions. However, phage Ar*π*, which we morphologically classified as a member of* Siphoviridae*, is only active in a narrow pH range (6.0–8.0).

Bacteriophage SIOΦ had the highest percent adsorption to the* Bacillus* host (50%) and released the largest number of particles (~74) from host cells (Table 4) and its phage titer was the highest (10^13^) (Table 2). Due to high activity at low and high temperature and pH, SIOΦ seems to be the best candidate for use in industry.

The literature contains many descriptions of experiments conducted under different conditions and hence with varying results. Some* Bacillus subtilis* phages such as SP02c1 or SP82 have a higher percent of adsorption than our phages [61] while, on the other hand, phage SPO1 has the same burst size as SIOΦ [62].

At least one of the characterized isolated bacteriophages was lytic for each tested strain of* Bacillus subtilis*. Thus, they could be suitable for the phagotyping of this bacterial species.

## Figures and Tables

**Table 1:** *Bacillus subtilis* strains and bacteriophages.

Number	Strain	Origin	Bacteriophage typing			
			SUB*ω*	SPO*σ*	SIOΦ	AR*π*
1	*B. subtilis *ATCC 6633	Polish Academy of Sciences, Wroclaw	+	+	−	−

2	*B. subtilis *168	Institute, Rijksuniversiteit Groningen, Holland	0	+	0	0
3	*B. subtilis *SWV215		+	+	−	−

4	*B. subtilis *10	University of Wroclaw, Faculty of Biotechnology	−	−	+	−

5	*B. subtilis *B3	Wroclaw University of Environmental and Life Sciences, Faculty of Food Science	+	+	−	+
6	*B. subtilis* Ż7-mutant B3		+	+	−	+
7	*B. subtilis* Ż17-mutant B3		+	+	−	+
8	*B. subtilis* Ż25-mutant B3		+	+	−	+
9	*B. subtilis* Hb36-mutant B3		+	+	−	+
10	*B. subtilis* B20		0	+	−	0
11	*B. subtilis* P22		+	+	−	+
12	*B. subtilis* KT20		0	−	0	0
13	*B. subtilis* 172		+	+	−	+
14	*B. subtilis* B24		0	+	−	0

15	*B. subtilis* PCM1938	Polish Academy of Sciences, Wroclaw	+	+	−	−
16	*B. subtilis* PCM 2226		+	+	+	−
17	*B. subtilis* PCM 486		0	+	−	0
18	*B. subtilis* PCM 2189		+	+	−	−
19	*B. subtilis* PCM 2005		+	+	−	0
20	*B. subtilis* PCM 2224		+	+	−	−

(−) Plaques not formed, (+) distinct clear plaques, and (0) hazy plaques.

**Table 2:** Phage titers (pfu/mL) for *B. subtilis* strains.

Phage	SIOΦ	SUB*ω*	SPO*σ*	AR*π*

*Bacillus *strain	*B. subtilis* 10	*B. subtilis* ATCC 6633	*B. subtilis* SWV215	*B. subtilis* B3

Phage titer (pfu/mL)	10^13^	10^10^	10^10^	10^12^

**Table 3:** Particle sizes of SIOΦ, SUB*ω*, SPO*σ*, and AR*π* phages.

Host	Phage	Head diameter [nm × nm]	Tail length [nm]
*B. subtilis* 10	SIOΦ	100 × 90	208

*B. subtilis* ATCC 6633	SUB*ω*	67 × 67	223

*B. subtilis* SWV215	SPO*σ*	91 × 87	70

*B. subtilis* B3	AR*π*	41 × 47	342

**Table 4:** Biological characteristics of SIOΦ, SUB*ω*, SPO*σ*, and AR*π* phages.

Phage	Adsorption [%]	Burst size [pfu/mL]	Latent period [min]	Growth time [min]
SIOΦ	50	∼74	55–65	40
SUB*ω*	12	∼8	55–65	30
SPO*σ*	7.5	∼23	75–85	40
AR*π*	12	∼37	55–65	30
